# A New Destroyer of Hair

**Published:** 1874-01

**Authors:** 


A New Destroyer of the Hair.—Under the above title Dr. Bœttger, in the *Memerobilien,* says that we possess a new material for destruction of hair, of a most suitable description, in a mixture of one part of crystalized sulphydrate of sodium with three parts of fine carbonate of lime mixed and reduced to a very fine powder. This mixture may be kept any length of time without alteration in well-closed bottles. When moistened with a drop of water and laid by means of the back of a knife on the part of the skin covered with hair, we in a few minutes find the thickest hair turned into a soft mass, easily removed by means of water. If it remain on the part long it will cause a slight irritation of the skin.—*London Medical Record.*

